# Characterization of the Lytic Capability of a LysK-Like Endolysin, Lys-phiSA012, Derived from a Polyvalent *Staphylococcus aureus* Bacteriophage

**DOI:** 10.3390/ph11010025

**Published:** 2018-02-24

**Authors:** Jumpei Fujiki, Tomohiro Nakamura, Takaaki Furusawa, Hazuki Ohno, Hiromichi Takahashi, Junya Kitana, Masaru Usui, Hidetoshi Higuchi, Yasunori Tanji, Yutaka Tamura, Hidetomo Iwano

**Affiliations:** 1 Laboratory of Biochemistry, School of Veterinary Medicine, Rakuno Gakuen University, Ebetsu 069-8501, Japan; j-fujiki@rakuno.ac.jp (J.F.); tomohiro-tobi-@hotmail.co.jp (T.N.); s21441012@stu.rakuno.ac.jp (T.F.); leafmoon-0812@honey.ocn.ne.jp (H.O.); l3ump_fnch@yahoo.co.jp (H.T.); s21361043@stu.rakuno.ac.jp (J.K.); 2 Laboratory of Food Microbiology and Food Safety, School of Veterinary Medicine, Rakuno Gakuen University, Ebetsu 069-8501, Japan; usuima@rakuno.ac.jp (M.U.); tamuray@rakuno.ac.jp (Y.T.); 3 Laboratory of Veterinary Hygiene, School of Veterinary Medicine, Rakuno Gakuen University, Ebetsu 069-8501, Japan; higuchi@rakuno.ac.jp; 4 Department of Bioengineering, Tokyo Institute of Technology, Yokohama 226-8502, Japan; ytanji@bio.titech.ac.jp; 5 Center for Veterinary Drug Development, Rakuno Gakuen University, Ebetsu 069-8501, Japan

**Keywords:** antibiotic resistant, multidrug resistant, antimicrobial agent, phage therapy, bacteriophage, endolysin, staphylococci, *Staphylococcus aureus*

## Abstract

Antibiotic-resistant bacteria (ARB) have spread widely and rapidly, with their increased occurrence corresponding with the increased use of antibiotics. Infections caused by *Staphylococcus aureus* have a considerable negative impact on human and livestock health. Bacteriophages and their peptidoglycan hydrolytic enzymes (endolysins) have received significant attention as novel approaches against ARB, including *S. aureus*. In the present study, we purified an endolysin, Lys-phiSA012, which harbors a cysteine/histidine-dependent amidohydrolase/peptidase (CHAP) domain, an amidase domain, and a SH3b cell wall binding domain, derived from a polyvalent *S. aureus* bacteriophage which we reported previously. We demonstrate that Lys-phiSA012 exhibits high lytic activity towards staphylococcal strains, including methicillin-resistant *S. aureus* (MRSA). Analysis of deletion mutants showed that only mutants possessing the CHAP and SH3b domains could lyse *S. aureus*, indicating that lytic activity of the CHAP domain depended on the SH3b domain. The presence of at least 1 mM Ca^2+^ and 100 µM Zn^2+^ enhanced the lytic activity of Lys-phiSA012 in a turbidity reduction assay. Furthermore, a minimum inhibitory concentration (MIC) assay showed that the addition of Lys-phiSA012 decreased the MIC of oxacillin. Our results suggest that endolysins are a promising approach for replacing current antimicrobial agents and may contribute to the proper use of antibiotics, leading to the reduction of ARB.

## 1. Introduction

Antibiotic-resistant bacteria (ARB) have spread rapidly worldwide and are an important global health issue. ARB often result from the inappropriate use of antibiotics [1,2,3] and the occurrence of ARB is correlated with the amount of antibiotics used [4]. In the United States, about half of antibiotics are used to treat humans annually and the other half to treat livestock and for other agricultural applications [5]. Notably, antibiotics are administered to livestock for not only preventive and therapeutic approaches against infectious diseases, but also to improve growth [6]. Annual worldwide veterinary antibiotics (VA) use is now 10^5^–10^6^ tones [7,8]. Several reports have suggested that antibiotics are used more heavily in livestock than in humans and VAs may be the largest source of ARB [9,10,11]. Indeed, transmission of ARB, such as methicillin-resistant *Staphylococcus aureus* (MRSA), by veterinary staff in contact with ARB carrier animals, and the potential role of the farm environment in the spread of ARB, have been reported [12,13,14,15,16], suggesting the environmental transfer of ARB from animals to humans.

ARBs that exhibit resistance to more than three classes of antibiotics are called multidrug-resistant bacteria (MDR). *Staphylococcus aureus*, an ESKAPE pathogen (*Enterococcus faecium*, *Staphylococcus aureus*, *Klebsiella pneumoniae*, *Acinetobacter baumanii*, *Pseudomonas aeruginosa* and *Enterobacter* spp.), is frequently isolated from clinical samples and is considered the major MDR. *S. aureus* has a considerable negative impact on human and veterinary medicine because the treatment of antibiotic-resistant *S. aureus*, including MRSA infections, are complicated due to the bacterium′s multiple antibiotic-resistant mechanisms. In the veterinary field, mastitis caused by *S. aureus* negatively impacts milk production, leading to an economic loss of more than $100 million annually in the United States and Japan [17,18]. *S. aureus* also is the most common pathogen causing chronic mastitis [19]. Furthermore, MRSA is spreading in hospitalized patients [20]. According to World Health Organization (WHO) reports, patients with MRSA are estimated to be 64% more likely to die compared with patients with non-resistant infections. A study by Lord O’Neill and colleagues, commissioned by the United Kingdom government, estimated that ARB would cause 10 million deaths each year by 2050 and will become a bigger risk than cancer [21,22]. Therefore, we face an unprecedented challenge from the emergence of ARB such as MRSA and MDR *P. aeruginosa*, putting humans and livestock at great risk. A novel alternative antimicrobial strategy is thus urgently required. Bacteriophages and bacteriophage enzyme-based approaches have potential as alternative tools for overcoming infectious diseases caused by ARB [23,24].

Phage therapy, an alternative to classical antibiotic therapy, is receiving significant attention because bacteriophages are the most abundant organisms and infect bacteria specifically [24,25]. This allows their rapid and simple isolation from the environment and clinical application to kill pathogenic bacteria. We previously demonstrated that phage therapy is effective for treating equine keratitis caused by *P. aeruginosa* [26] and the lysis of antibiotic-resistant *P. aeruginosa* [27]. We also reported the isolation of a bacteriophage, phiSA012, and its wide host range towards various *S. aureus* strains [28] and its effective lytic capacity in a mouse mastitis model caused by *S. aureus* [29]. Challenges in the use of phage therapy have also been reported, such as the occurrence of phage-resistant bacteria, and horizontal gene transfer leading to increased bacterial virulence [30,31].

Endolysins are peptidoglycan hydrolytic enzymes produced by bacteriophages and cleave cell wall peptidoglycan. Endolysins can thus target bacteria at the last stage of the lytic cycle using mechanisms different from those of antibiotics, and show cell lysis activity toward ARB such as *S. aureus* [23] and *P. aeruginosa* [32] in the absence of horizontal gene transfer. Interestingly, bacteria are unlikely to develop resistance towards endolysins [33,34] because endolysins may cleave peptidoglycan sites essential for bacterial survival, and bacteriophages have developed highly efficient enzymes through evolution [35,36,37]. In addition, endolysins affect persisters, which are dormant bacteria showing high tolerance to antibiotics, and biofilms [38,39], and can be engineered to readily fuse with functional peptides [37]. Taken together, endolysins have enormous potential as a flexible tool for combatting ARB. 

*S. aureus* bacteriophage endolysins such as Lys-phiK, Lys-GH15 and Lys-phiTwort have been isolated and investigated [40,41,42]. These endolysins are multidomain enzymes composed of a cysteine, histidine dependent amidohydrolase/peptidase (CHAP) domain at the N-terminus, an amidase (AMID) domain and a SH3b cell wall binding domain at the C-terminus and demonstrate high lytic activity toward *S. aureus.* The lytic efficacy and immune response of Lys-GH15 in vivo was previously reported [41,43]. In addition, the structures of three individual domains of Lys-GH15 were determined [44]. Notably, Lys-phiK is regarded as one of the best-characterized endolysins [23] and reveals a broad antimicrobial spectrum toward staphylococci isolated clinically from bovine and human [40]. It was reported that the Lys-phiK CHAP domain plays a critical role in the lysis of live staphylococcus and that lysis depends on the SH3b domain [45]. The crystal structure of the CHAP domain has been determined [46]. However, the properties of Lys-phiK related endolysins are not fully characterized. The aim of the present study was to demonstrate the possible preventive and therapeutic utility of efficient endolysins for clinical applications. To this end, we isolated and purified Lys-phiSA012, which has high amino acid sequence similarity with Lys-phiK, and studied the properties of Lys-phiSA012 lytic activity.

## 2. Materials & Methods

### 2.1. Bacterial Strains

Bacterial strains used in the turbidity reduction assays, minimum inhibitory concentration (MIC) assay, and minimum bactericidal concentration (MBC) assay, are summarized in Table 1. The staphylococcal and streptococcal strains were grown at 37 °C with aeration in lysogeny broth (LB) containing 2% tryptone (Difco, Detroit, MI, USA), 0.5% yeast extract (Difco) and Tod-Hewitt broth (THB) (Kanto Kagaku, Tokyo, Japan). *Escherichia coli* DH5α and BL21(DE3) (Takara, Otsu, Japan) were grown at 37 °C with aeration in LB medium for all subcloning. When needed, 100 µg/mL ampicillin was added to the LB medium (final concentration).

### 2.2. Bacteriophage and Genome Analysis

We previously reported the isolation of phiSA012 from a sewage treatment plant located in Tokyo (Japan) [28]. The genomic sequence of phiSA012 was published (NC_023573.1) and the endolysin amino acid sequence is available (YP_009006722). Protein sequence analysis and classification of Lys-phiSA012 were performed using InterPro to predict the domain architecture. The DNA of phiSA012 was isolated as described by Synnott et al. using a plate assay and centrifuged in polyethylene glycol 6000-NaCl [28]. PhiSA012 was then incubated in SNET buffer (400 µg/mL ProteinaseK (Takara), 20 mM Tris-HCl, 400 mM NaCl, 1% SDS, 5 mM EDTA) at 55 °C overnight, phiSA012 DNA was extracted using phenol and chloroform, then purified by ethanol precipitation.

### 2.3. Purification of Lys-phiSA012 Recombinant Protein

The extracted phiSA012 DNA was used for subcloning of Lys-phiSA012. The endolysin gene was amplified with the primers Lys012-1Fw and Lys012-495Rv, as shown in Table 2. The amplified fragment was purified and subcloned into pGEX-6P-2 (GE, Buckinghamshire, UK) encoding a glutathione S-transferase (GST)-tag sequence at the C-terminal using an In-Fusion HD cloning kit (Clontech, Palo Alto, CA, USA), then the plasmid encoding Lys-phiSA012, pGEX-Lys012WT, was constructed. All subcloning was performed in *E. coli* DH5α. Plasmid construct accuracy was confirmed by DNA sequence analysis using a Big Dye Terminator V3.1 cycle sequencing kit (Applied Biosystems, Foster City, CA, USA) and an Applied Biosystems 3130 Genetic Analyzer. The cloned gene expression was performed as described elsewhere [45,50] with slight modifications. In brief, the plasmid was used to transform *E. coli* BL21(DE3) and expression of the endolysin gene was induced by the addition of 0.1 mM isopropyl-β-thiogalactopyranoside (final concentration) (Nacalai Tesque, Kyoto, Japan). Cells were incubated with shaking overnight at 25 °C in a BIO-SHAKER BR-40LF (Taitec, Saitama, Japan). After centrifugation at 2300× *g* for 5 min at 4 °C, the supernatant was removed and 50 mM Tris-HCl, 1 M MgCl_2_ and 10% NP-40 (Wako, Osaka, Japan) were added to the pellet and the mixture was sonicated using a Bioruptor UCD-200 (Cosmo Bio, Tokyo, Japan). The mixture was centrifuged at 16,000× *g* for 30 min at 4 °C, then the supernatant containing soluble protein was purified using Econo-Pac^®^ disposable chromatography columns (Bio-Rad Laboratories, Inc., Hercules, CA, USA) packed with glutathione Sepharose 4B (GE). The identity and concentration of the expressed protein was confirmed by sodium dodecyl sulfate polyacrylamide gel electrophoresis (SDS-PAGE). The obtained Lys-phiSA012 was stored at −30 °C until use.

### 2.4. Generation of Domain(s) Deletion Mutants

pGEX-Lys012WT was used for the construction of seven deletion mutant plasmids encoding a single domain or multiple domains of endolysin. Certain fragments of gene for Lys-phiSA012 were amplified with the primers summarized in Table 2. To construct pGEX-Lys012Δmt2, two amplified fragments, corresponding to amino acids 1–221 and 390–495, were used for overwrap PCR using Lys012-1Fw and Lys012-495Rv primers. The amplified fragments were subcloned into pGEX-6P-2 and each protein was purified as described above.

### 2.5. Turbidity Reduction Assays

The lytic activity of Lys-phiSA012 and each deletion mutant protein was assessed using turbidity reduction assays as described by Becker et al. [45] and Son et al. [51], with some modifications. In brief, staphylococcal strains and streptococcal strains were grown in LB medium or THB medium at 37 °C to an OD_600_ of 1.0. Each culture was centrifuged at 2300× *g* for 5 min at 4 °C and the cells were resuspended in 2× LB medium, then stored on ice until use. Turbidity reduction assays were initiated by adding the same amount of purified Lys-phiSA012 or each domain(s) deletion mutant, then the OD_600_ value was monitored using a plate reader (Sunrise Rainbow Thermos RC, TECAN Austria GmbH, Salzburg, Austria). To evaluate the effect of Ca^2+^ and Zn^2+^ on endolysin lytic activity, SA003 was prepared as described above. After centrifugation at 2300× *g* for 5 min at 4 °C, the cells were resuspended in TBS (50 mM Tris-HCl, 138 mM NaCl, 2.7 mM KCl) buffer with CaCl_2_ (0 µM, 1 µM, 10 µM, 100 µM, 1 mM, 2.5 mM, 5 mM) or ZnCl_2_ (0 µM, 1 µM, 10 µM, 100 µM, 500 µM, 1 mM, 2.5 mM). The OD_600_ value was monitored with incubation using the plate reader after the addition of Lys-phiSA012.

### 2.6. MIC and MBC Assays

MIC and MBC assays were performed as described previously [45,52] with some modifications. The MIC values of oxacillin with or without Lys-phiSA012 towards SA003 were determined in 96-well plates. Each plate incubated serial 2-fold dilution of oxacillin and Lys-phiSA012 using LB medium overnight at 37 °C at concentrations ranging from 64 to 0 µg/mL and from 2.0 to 0 µg/mL, respectively. Overnight incubation was initiated by adding 10 µL SA003 to each well of the 96-well plate that also contained 100 µL LB medium. The MBC values of Lys-phiSA012 towards SA003 with 25% glycerol were determined using LB medium after overnight incubation at 37 °C. After the MIC assay of Lys-phiSA012 with 25% glycerol, 10 µL of liquid from each well in the 96-well plate was transferred to a fresh 96-well plate containing 100 µL antimicrobial agent free LB medium. The MIC and MBC values were evaluated by visual inspection after overnight incubation. MIC and MBC assays of glycerol were also performed using the same methods.

### 2.7. Bioinformatics Analysis

Amino acid sequences for multiple alignment and protein structure homology modeling were obtained from the National Center for Biotechnology Information (NCBI) database. Multiple alignment analysis between endolysin sequences was performed using GENETYX software v.10. The 3D protein structure models of the cysteine, histidine dependent amidohydrolases/peptidases (CHAP) and amidase (AMID) domain on Lys-phiSA012 were constructed and analyzed with modeling by homology to the existing crystal structure using the SWISS-MODEL server [53,54,55,56]. Homologous proteins and amino acid sequence alignments are summarized in Table 3 and Supplementary Materials Figure S1. A BLAST search was performed using the nucleotide sequence of Lys-phiK obtained from NCBI database as a query sequence. Endolysins which revealed over 90% query cover and identity are summarized in Table 4.

### 2.8. Statistical Analysis

Statistical analysis was conducted using the Tukey-Kramer test based on one-way ANOVA from three independent experiments. Statistically significant differences are indicated by asterisks (*: *p* < 0.05, **: *p* < 0.01) or crosses (^†^: *p* < 0.05).

## 3. Results

### 3.1. Lytic Activity and the Antimicrobial Spectrum of Lys-phiSA012.

**Figure 1:**
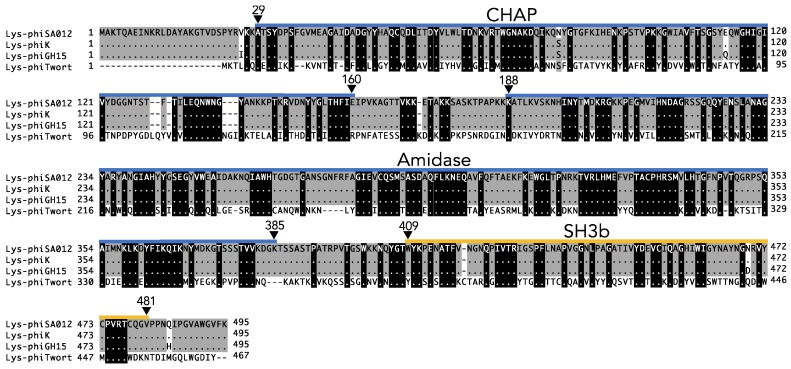
Prediction of the domain architecture and multiple alignment analysis of Lys-phiSA012. Lys-phiSA012 is composed of a CHAP, AMID, and SH3b domain. The amino acid sequence of Lys-phiSA012 was analyzed using InterPro. Blue and yellow lines indicate the domain structure of Lys-phiSA012. Multiple alignment analysis of the full length endolysins Lys-phiSA012, Lys-phiK, Lys-phiGH15, and Lys-phiTwort. Identical residues are shown as dots and amino acid residues conserved between the four endolysins are highlighted by a black (100%) or gray (80%) scale.

The endolysin of phiSA012, Lys-phiSA012, is encoded in ORF51 and is composed of 495 amino acids. The conserved domain of Lys-phiSA012 was shown by bioinformatics analysis to be a cysteine, histidine dependent amidohydrolase/peptidase (CHAP) domain at the N-terminus encompassing amino acid residues 29–160, an amidase (AMID) domain encompassing amino acid residues 188–385, and a SH3b cell wall binding domain at the C-terminus encompassing amino acid residues 409–481 (Figure 1). 

**Figure 2:**
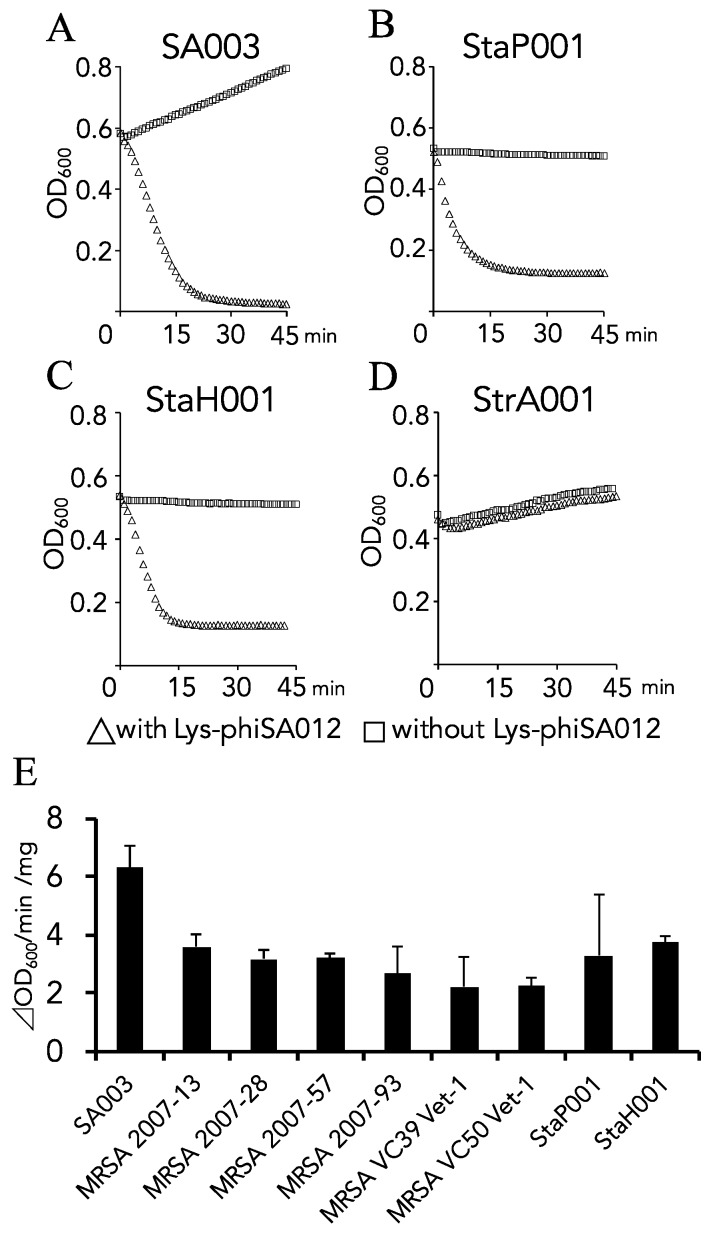
Antimicrobial spectrum of Lys-phiSA012. Lytic activities towards SA003, StaP, StaH, StrA and MRSA were examined using a turbidity reduction assay, as described in the materials and methods section. SA003 was isolated from bovine milk and has been reported to be susceptible to phiSA012 [28]. SA003 was also used in a mouse mastitis model [29]. MRSA strains were isolated from veterinarians and staff members of veterinary clinics [48,49]. Lys-phiSA012 (**A**) 109 µg/mL, (**B**) 132 µg/mL, (**C**) 132 µg/mL and (**D**) 106 µg/mL was added to the bacterial cultures. The values of OD_600_ were plotted each minute. (**E**) Lytic activities towards staphylococcal strains were examined. The maximum rate for each reaction is represented as ∆OD_600_ min^−1^ mg^−1^, calculated as previously reported [61] and presented as means ± standard deviation (SD).

**Figure 3:**
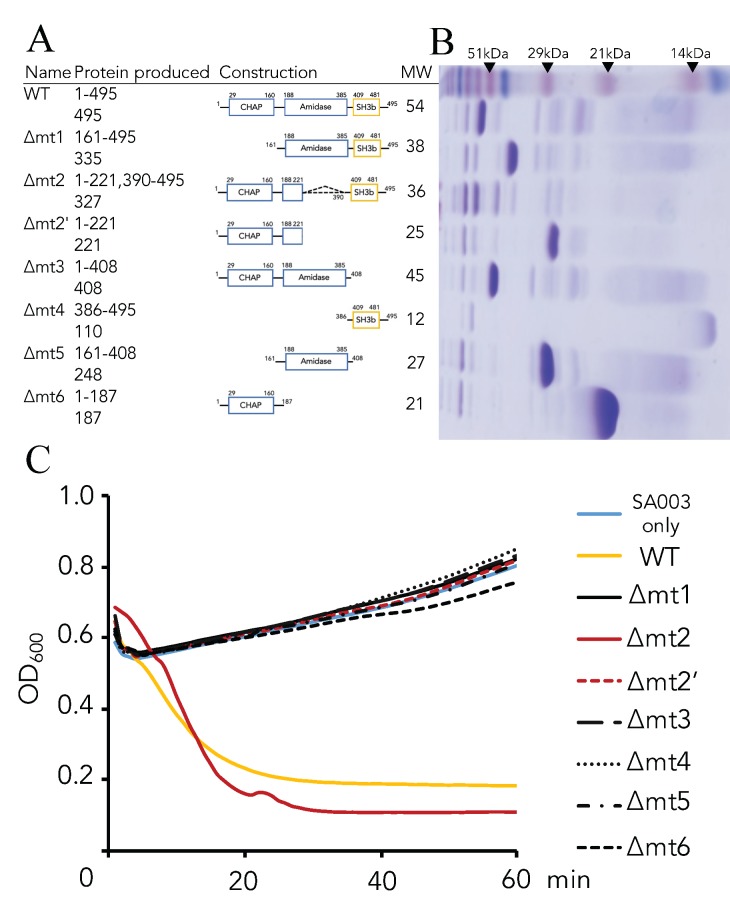
Schematic representation of Lys-phiSA012 and domain(s) deletion mutants and their lytic activities towards SA003. (**A**) Schematic representation of the Lys-phiSA012 protein construct. Amino acid residues 29–160: CHAP domain, 188–385: AMID domain, 409–481: SH3b domain. (**B**) SDS-PAGE of purified Lys-phiSA012 and each domain(s) deletion mutant, performed using 15% polyacrylamide gel. Upper lane; Standard molecular weight markers. (**C**) Lytic activities of Lys-phiSA012 and each domain(s) deletion mutant towards SA003. Except for ∆mt2, 163.8 pmol of the endolysin was added to the culture medium. For ∆mt2, 45.98 pmol was added.

Comparison of the amino acid sequences of Lys-phiSA012 with the characterized *Staphylococcus* endolysins, Lys-phiK, Lys-phiGH15, and Lys-phiTwort, revealed that Lys-phiSA012 has high amino acid sequence similarity with Lys-phiK (99.80%) and Lys-phiGH15 (98.99%), but not with Lys-phiTwort (44.54%) (Figure 1). Furthermore, endolysins which have high nucleotide sequence similarity with Lys-phiK (LysK-like eondolysins) were found by a BLAST search, as summarized in Table 4; however, the lytic capabilities of LysK-like endolysins (with the exception of Lys-GH15) remain poorly characterized. Previous reports have focused on the lytic capabilities of the bacteriophages vB_Sau_CG, vB_Sau_Clo6, vB_Sau_S24 [57], S25-3 [58], MCE-2014 [59] and IPLA-RODI [60], from which the LysK-like endolysins are derived, or only genome sequences of bacteriophages JD007, S25-4, SA3 and qdsa002 have been registered. Therefore, we initially produced Lys-phiSA012WT and performed turbidity reduction assays to confirm the lytic activity of Lys-phiSA012. As shown in Figure 2A, Lys-phiSA012 showed high lytic activity towards SA003 (A schematic of the produced Lys-phiSA012WT protein and SDS-PAGE analysis of the purified protein are shown in Figure 3A,B). Broad lytic activity of phiSA012 and Lys-phiK has been reported [28,40]. To determine whether Lys-phiSA012 similarly has lytic activity beyond *S. aureus*, we examined the antimicrobial spectrum of Lys-phiSA012 towards staphylococcal strains and *Streptococcus agalactiae*. Lys-phiSA012 showed high antimicrobial activity towards not only SA003, but also StaP001 and StaH001 as expected. In addition, the OD_600_ was reduced within a few minutes of adding Lys-phiSA012, with an initial OD_600_ of 0.4–0.6 being reduced to OD_600_ <0.2 over 10 min (Figure 2A–C). 

However, Lys-phiSA012 could not lyse StrA001, as shown by the turbidity reduction assay, and the initial OD_600_ increased with increasing incubation time (Figure 2D). We next assessed the antimicrobial activity of Lys-phiSA012 towards MRSA strains resistant against more than four antibiotics (Table 1). The turbidity reduction assay showed lytic activity of Lys-phiSA012 towards six MRSA strains (Figure 2E). The maximum rate for the lysis of SA003 (∆OD_600_ min^−1^ mg^−1^) was higher than that for the other strains.

### 3.2. Critical Region of the Lys-phiSA012 Protein Required for Lytic Activity 

Next, we constructed plasmids encoding Lys-phiSA012 domain(s) deletion mutants to clarify the region of Lys-phiSA012 constructs critical for lytic activity towards SA003. The purified proteins were identified by SDS-PAGE analysis (Figure 3B). Turbidity reduction assays using the domain(s) deletion mutants clearly demonstrated that Lys-phiSA012∆mt2 showed high lytic activity similar to Lys-phiSA012WT (Figure 3C) whereas the other mutants showed no lytic activity towards SA003.

### 3.3. Optimal Ca^2+^ and Zn^2+^ Concentration for the Lytic Activity of Lys-phiSA012

**Figure A1:**
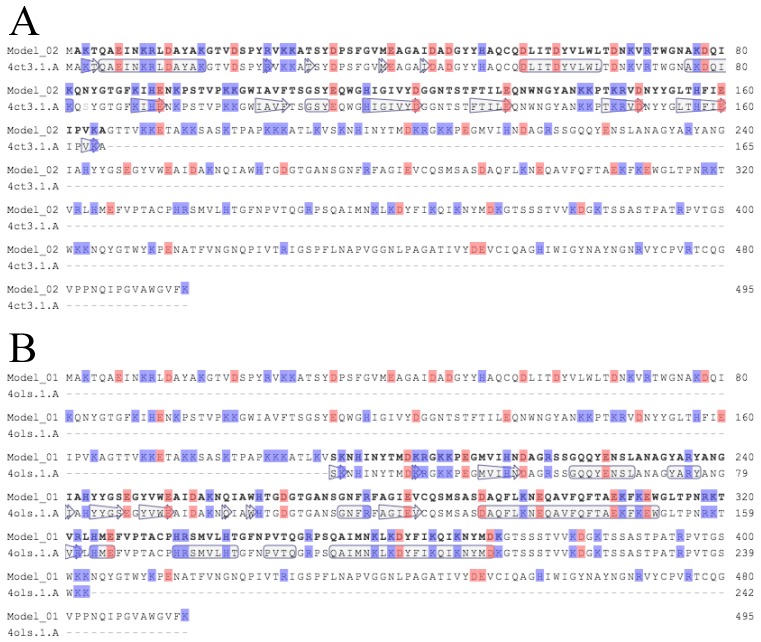
Model-template alignment in the protein structure model. Circles and arrows represent alpha-helices and beta-strands, respectively. Blue: positively charged amino acids, red: negatively charged amino acids. (**A**) Model-template alignment of the CHAP domain. Model_02; Lys-phiSA012 sequence. 4act. 1. A; Lys-phiK sequence. Lys-phiSA012 and Lys-phiK alignments of the other domains are described in Figure 1. (**B**) Model-template alignment of the AMID domain. Model_01; Lys-phiSA012 sequence. 4ols. 1. A; Lys-phiGH15 sequence. Lys-phiSA012 and Lys-phiGH15 alignments of the other domains are described in Figure 1. These data were analyzed using the SWISS-MODEL server.

Deletion analysis demonstrated that a CHAP domain and a SH3b domain play important roles for lytic activity. Enhancement of the lytic activity of endolysins by ions was previously reported [45,50]. Therefore, we next investigated the presence of an ion binding site in the Lys-phiSA012 catalytic domains. 3D models of the CHAP and AMID domain structures were constructed using the SWISS-MODEL server. The homologous proteins used in structure modeling are summarized in Table 3 and the model-template alignment is shown in Figure A1. 

**Figure 4:**
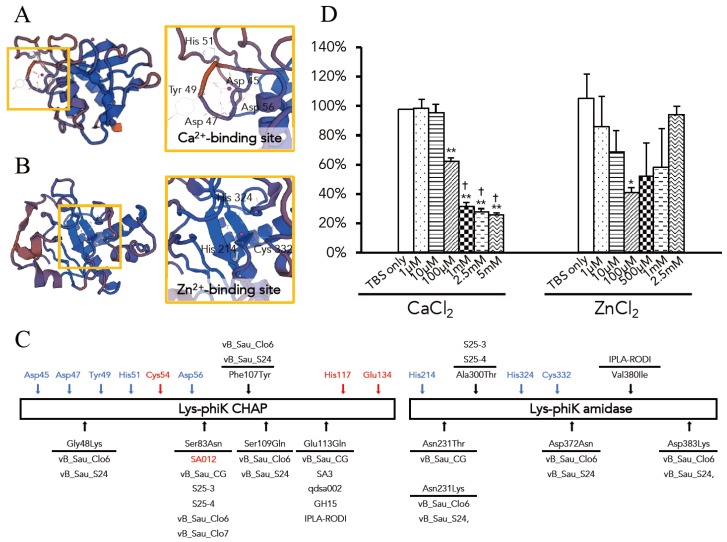
Protein structure modeling of the CHAP and AMID domains, and the effects of Ca^2+^ and Zn^2+^ on the lytic activity of Lys-phiSA012. Protein structure models of (**A**) the CHAP and (**B**) AMID domains in Lys-phiSA012. The models were constructed using the SWISS-MODEL server and the homologous proteins used for protein structure modeling are summarized in Table 3. Detailed views of the Ca^2+^ and Zn^2+^ coordination sites are framed in yellow. The Ca^2+^-binding site is composed of Asp45, Asp47, Tyr49, His51 and Asp56. The Zn^2+^-binding site is composed of His214, His324 and Cys332. Ions are shown as pink spheres. (**C**) Schematic image of the CHAP and AMID domain of Lys-phiK. Blue arrows indicate the amino acid residues which contacts with Ca^2+^ and Zn^2+^. Red arrows indicate the active site residues of the CHAP domain. Black arrows indicate the amino acid changes in LysK-like endolysins summarized in Table 4. Bacteriophages whose endolysins are related to Lys-phiK are also indicated. Lys-phiSA012 differs by single amino acid residue from Lys-phiK, Ser83Asn on a CHAP domain. (**D**) The effects of Ca^2+^ and Zn^2^ on the lytic activity of Lys-phiSA012 were analyzed using a turbidity reduction assay as described in the materials and methods section. Data are shown as relative OD_600_ reduction rate (initial OD600 divided by OD600 60 min after adding Lys-phiSA012). The values represent means ± SD. Significance was analyzed by the Tukey-Kramer test based on one-way ANOVA and is indicated by asterisks compared to TBS alone (*: *p* < 0.05, **: *p* < 0.01) or crosses against 100 µM Ca^2+^ (^†^: *p* < 0.05).

The constructed model of the CHAP domain revealed that Ca^2+^ is bound in the N-terminal region and interacts with the side chains of Asp45, Asp47, Tyr49, His51 and Asp56 (Figure 4A). In addition, Cl^-^ and Hg^2+^ were indicated as bound ligands for the CHAP domain (Table 3). The constructed model of the AMID domain showed that Zn^2+^ is bound in the center of the domain and interacts with the side chains of His214, His324 and Cys332 (Figure 4B), and Mg^2+^ and Fe^3+^ were indicated as bound ligands for the AMID domain (Table 3). As shown in Figure 4C, no amino acid changes were observed in LysK-like endolysins at the Ca^2+^ and Zn^2+^-binding site residues or at the CHAP active site residues of Lys-phiK [62].

**Figure A2:**
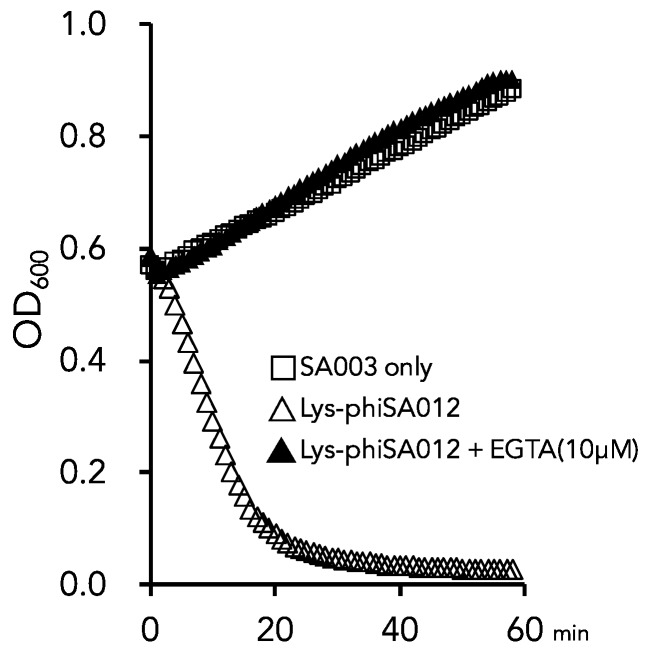
The effect of EGTA treatment on Lys-phiSA012 lytic activity. The lytic activity of Lys-phiSA012 w/wo EGTA was determined using the turbidity reduction assay. SA003 was grown in LB medium w/wo 10 mM EGTA and the assays were initiated by the addition of 64 µg/mL Lys-phiSA012.

It was reported that Zn^2+^ inhibits the Lys-phiK activity and that Ca^2+^ enhances the stability of Lys-phiK but has no effect on lytic activity [63]. However, ions in the Lys-phiK buffer were not removed in those assays. Sanz-Gaitero et al. also commented on this point and suggested that Zn^2+^ might play a regulatory role [46]. Therefore, we examined the effects of Ca^2+^ and Zn^2+^ on the lytic activity of Lys-phiSA012 to determine the optimal Ca^2+^ and Zn^2+^ concentrations. Turbidity reduction assays were performed in the presence and absence of Ca^2+^ or Zn^2+^ in TBS buffer. The addition of Ca^2+^ significantly reduced the OD_600_ in a dose dependent manner. Notably, a high concentration of Ca^2+^ (above 1 mM) significantly increased Lys-phiSA012 activity compared to the addition of 100 µM Ca^2+^ (Figure 4C). Lys-phiSA012 exhibited no lytic activity in the absence of Ca^2+^ and the OD_600_ value did not change from the initial value of OD_600_ 0.6. The addition of 100 µM Zn^2+^ resulted in a significant decrease in OD_600_ compared to TBS alone; interestingly, however, the addition of 1 µM Zn^2+^ did not enhance the lytic activity of Lys-phiSA012 compared to TBS alone. We further examined whether divalent ions in LB medium affect lytic activity. Lys-phiSA012 in LB medium containing 10 µM EGTA did not inhibit the growth of SA003 (Figure A2).

### 3.4. MIC Assays of Oxacillin w/wo Lys-phiSA012

**Figure 5:**
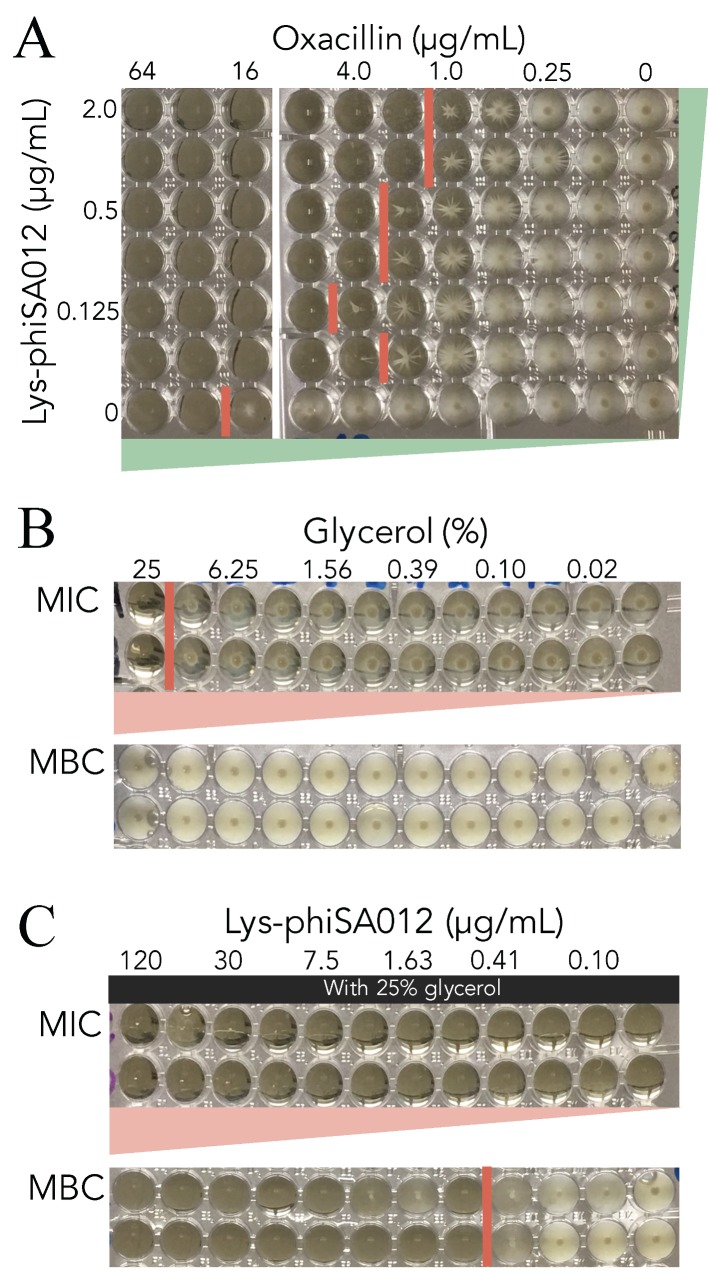
MIC and MBC assays. MIC and MBC assays were performed in 96-well plates. Red lines indicate the border of bacterial growth. (**A**) MIC assay of oxacillin towards SA003 with or without Lys-phiSA012. (**B**) MIC and MBC assays of glycerol towards SA003. (**C**) MIC and MBC assays of Lys-phiSA012 with 25% glycerol towards SA003.

The MIC of oxacillin towards SA003 with or without (w/wo) Lys-phiSA012 was determined to confirm whether Lys-phiSA012 acts cooperatively with antibiotics due to the different mechanisms underlying the antimicrobial activities of endolysins and antibiotics. The oxacillin MIC without Lys-phiSA012 was ≥32 µg/mL, whereas the addition of 1.0 µg/mL or 2.0 µg/mL Lys-phiSA012 reduced the oxacillin MIC 16-fold (Figure 5A).

Next, we examined suitable vehicles for endolysins. As shown in Figure 5B, 25% glycerol inhibited SA003 growth in MIC assays due to its bacteriostatic (and not bactericidal) activity, given that bacterial growth resumed after subculture, as confirmed by MBC assays (Figure 5B). An accurate MIC for Lys-phiSA012 could not be determined due to the initial high lytic activity of endolysins, as shown in Figure 2A–D and 3C, and their subsequent inactivation during overnight incubation. However, Lys-phiSA012 in 25% glycerol inhibited bacterial growth as shown by MIC assay after overnight incubation. Furthermore, the MBC of Lys-phiSA012 in 25% glycerol decreased at ≥0.815 µg/mL (Figure 5C).

## 4. Discussion

The discovery of penicillin by Alexander Fleming in 1928 lead to the purification and development of antibiotics in the 1940s, called the beginning of the era of antibiotics. The prevailing feeling during this era was that bacterial infectious diseases would be overcome by the development of various antibiotics. However, these antibiotics are no longer as effective as they were 70 years ago [5]. Furthermore, Gram-negative pathogens resistant to colistin, the last resort antibiotic against MDR, have been reported [64,65]. On the other hand, a personalized bacteriophage-based treatment approved by the Food and Drug Administration (FDA) of the United States as an emergency investigational new drug (eIND) was administered to a 68-year-old patient infected with multidrug-resistant *Acinetobacter baumannii* and resulted in dramatic improvement from a comatose state, despite the patient not responding to several antibiotics, including colistin [66]. In addition, the efficient lytic capability of endolysins towards MRSA in an in vivo assay has been reported [43,67,68]. These data suggested that the therapeutic use of bacteriophages could be applicable to similar cases, and endolysins might have the same potential as bacteriophages.

The present study has clearly demonstrated that Lys-phiSA012 shows high lytic activity towards *S. aureus,* including MRSA strains. The OD_600_ was reduced within a few minutes of adding Lys-phiSA012, and bacterial growth was clearly inhibited in the turbidity reduction assay, as it was previously shown for Lys-phiK [69]. Lys-phiSA012 also showed lytic activity towards *S. pseudintermidius* and *S. haemoliticus* (Figure 2A–C) but not towards *Streptococcus agalactiae* (Figure 2D), indicating that it has a specific antimicrobial spectrum limited to staphylococci, including MDR *S. aureus*. Specific host ranges of bacteriophages and endolysins could be advantageous for clinical applications as they would not disrupt our normal microbiota. Broad host ranges of bacteriophage SA012 [28], bacteriophage K [70] and bacteriophage Twort [71] towards *S. aureus* strains have been reported but the amino acid sequences of their endolysins showed incomplete identity. In particular, the sequence similarity between Lys-phiSA012 and Lys-phiTwort is less than 50% (Figure 1C), suggesting that endolysins have evolved to have highly diverse activities, allowing us to select the most effective and specific endolysin for combatting a specific bacterial strain. Further evaluation of Lys-phiSA012 efficacy in vivo is needed prior to its clinical application. In addition, Lys-phiSA012 showed high lytic activity within a few minutes, as shown in Figure 2A–C, which might lead to the rapid release of toxins from lysed bacterial cells. Therefore, it may be necessary to develop extended release Lys-phiSA012 that kill bacteria gradually in the body.

The recognition of peptidoglycans by endolysins determines their spectrum of activity and is regulated by a SH3b domain which binds a highly specific bacterial ligand [72]. In addition, the maximum lytic activities of several endolysins, such as Lys-phiK [45], LytA [73], and lysostaphin [74], depend on a SH3b domain. As shown in Figure 3C, Lys-phiSA012WT and ∆mt2, both containing CHAP-SH3b, showed maximum lytic activity towards SA003, suggesting that the Lys-phiSA012 SH3b domain is required for catalytic activity of the CHAP domain and plays a critical role in cell lysis. In addition, ∆mt3, which contains AMID-SH3b, showed no lytic activity, indicating that the AMID domain of Lys-phiSA012 may not play an essential role in lytic activity towards SA003 as assessed using turbidity reduction assays, or that deletion of this domain might cause conformational changes leading to defective lytic activity. These data are consistent with previous reports regarding various staphylococcal endolysins harboring a SH3b domain [73,75].

A Ca^2+^-binding site in the CHAP domain and a Zn^2+^-binding site in the AMID domain of Lys-phiSA012 were identified by bioinformatics analysis (Figure 4A,B). We estimated that the presence of Zn^2+^ might not effect lytic activity significantly because ∆mt3 might not efficiently lyse bacterial cell wall peptidoglycan; however, high lytic activity was observed in the presence of 100 µM Zn^2+^ (Figure 4C). It was reported that the CHAP domain of Lys-phiK, which has 99.39% amino acid sequence identity (Table 3, Figure A1A) with that of Lys-phiSA012, also harbors a Zn^2+^-binding site [64] based on structural analysis. We suggest that Zn^2+^ affects not only the AMID domain but also the CHAP domain, leading to potent lytic activity. Furthermore, Donovan et al. [73] and Becker et al. [50] provided clues for the relationship between the catalytic roles of the CHAP and AMID domains, suggesting that endolysins harboring dual lytic domains derived from staphylococcal phages might respond differently (i.e., either exolytic or endolytic) to bacterial peptidoglycan. Here, we demonstrated that divalent ions in the medium play a critical role in the lytic activity of Lys-phiSA012 (Figure A2), and maximum lytic activity was observed in the presence of at least 1 mM Ca^2+^, consistent with the extracellular concentration of Ca^2+^ which is closely regulated at around 1.2 mM [76], and 100 µM Zn^2+^, consistent with the intracellular concentration of Zn^2+^ [77]. Taken together, we suggest that Lys-phiSA012 is activated by divalent ions, especially Ca^2+^ and Zn^2+^, at specific minimum concentrations, binding to a specific site in the catalytic domains. As we known, the present study is the first to characterize the optimal range of Ca^2+^ and Zn^2+^ concentrations for the lytic activity of Lys-phiK and LysK-like endolysins. Changes in the amino acid residues in the CHAP and AMID domain among Lys-phiK, Lys-phiSA012 and other LysK-like endolysins (Table 4) are not located at the Ca^2+^-, Zn^2+^-binding sites (Figure 4C), suggesting that at least 1 mM Ca^2+^ and 100 µM Zn^2+^ might be required for efficient Lys-phiK and LysK-like endolysin lytic activity. Further investigations are needed to clarify the relationship between amino acid change(s) and the lytic activity of LysK-like endolysins compared to the lytic activity of Lys-phiK. In particular, further studies of the role of Ser83Asn found in more than half of LysK-like endolysins, including Lys-phiSA012 (Table 4, Figure 3C), are required because it was previously reported that certain single amino acid changes in the CHAP domain of Lys-phiK enhance lytic activity toward *S. aureus* stains [78].

It was reported that the CHAP domain of Lys-phiK cleaves at the pentaglycine linkage between peptidoglycan chains and that the AMID domain of Lys-phiK cleaves between N-acetylmuramic acid and L-alanine [45,79]. The mechanism underlying cell wall peptidoglycan digestion is different between endolysins and other antibiotics, and thus we tested the efficacy of Lys-phiSA012 with oxacillin towards *S. aureus*. As shown in Figure 5A, endolysins have significant potential for reducing antibiotics use because Lys-phiSA012 decreased the MIC of oxacillin more than 16-fold. In addition, the bactericidal activity of Lys-phiSA012 was shown to be limited in the presence of 25% glycerol, which we used as a vehicle for Lys-phiSA012 (Figure 5C). Notably, it was previously reported that MIC of Lys-phiK towards *S. aureus* is 78 ± 20 µg/mL [45]. A large amount of Lys-phiK might be required to kill these bacteria completely due to the inactivation of endolysin during overnight incubation in MIC assays; however, Lys-phiSA012 (≥0.815 µg/mL) with 25% glycerol clearly inhibited bacterial growth in the present study, suggesting that the combination of Lys-phiSA012 with bacteriostatic agents might enhance and prolong the lytic activity of endolysins.

Generally, endolysins can directly access and subsequently digest the cell wall peptidoglycans of Gram-positive bacteria, as shown in Figure 2A, but are largely ineffective towards Gram-negative bacteria due to the outer membrane barrier [25,34]. In addition, some bacteria, including *S. aureus,* can move into the intracellular spaces to escape the immune system, making antibiotics and endolysins ineffective because they cannot access the cytosol due to the cellular membrane [80]. However, endolysins can be engineered to overcome their limited activity towards Gram-negative and intracellular bacteria by being fused to outer membrane permeabilizing peptides, allowing their transfer across the membrane barrier [81,82,83]. Further investigations are needed to develop Lys-phiSA012 for use against intracellular *S. aureus*.

In conclusion, we purified a LysK-like endolysin, Lys-phiSA012, and demonstrated that its highly potent lytic activity towards staphylococci is not limited to *S. aureus*. Furthermore, our results suggest optimal conditions for Lys-phiK and LysK-like endolysins. Our results will contribute to the understanding of LysK-like endolysins and to promoting the development of alternatives for antibiotics, leading to the reduction of antibiotic-resistant bacteria and supporting the proper use of antibiotics.

## Figures and Tables

**Table 1:** Staphylococcal and streptococcal strains.

Bacterial Strains	Name	Refarences and Remarks				
*Staphylococcus aureus*	SA003	Synnott, A.J. et al. [28], Iwano, H. et al. [29]				
*Staphylococcus pseudointermedius*	StaP001	Field isolates identified by their 16s ribosomal RNA sequences.				
*Staphylococcus haemolyticus*	StaH001					
*Streptcoccus agalactiae*	StrA001					
		Scc *mec*	MLST	spa type	Antimicrobial-resistance pattern	Refarences
MRSA	MRSA 2007-13	II	NT	t002	MPIPC, GM, KM, EM, OTC, ERFX	Ishihara, K. et al. [47]
	MRSA 2007-28	II	NT	t1265	MPIPC, KM, EM,	
	MRSA 2007-57	IV	NT	t008	MPIPC, GM, KM, EM	
	MRSA 2007-93	II	NT	t062	MPIPC, KM, EM, OTC, CP, ERFX	
	MRSA VC39 Vet-1	IV	ST380	t021	MPIPC, SM, KM, GM, EM	Ishihara, K. et al.
	MRSA VC50 Vet-1	IV	ST30	t1852	MPIPC, KM, GM, EM, CPFX	[48]

MPIPC; oxacillin (breakpoint, 4 µg/mL), GM; gentamicin (16 µg/mL), KM; kanamycin (64 µg/mL), EM; erythromycin (8 µg/mL), OTC; oxytetracyclin (32 µg/mL), ERFX; enrofloxacin (4 µg/mL), CP; chloramphenicol (32 µg/mL) SM; streptomycin (64 µg/mL), CPFX; ciprofloxacin (4 µg/mL). Breakpoints were adopted according to the Clinical and Laboratory Standards Institute guidelines [49].

**Table 2:** Plasmids and primers.

Plasmids	Protein Produced (Amino Acids)	Forward Primes	Reverse Primers	Recipient Vectors
pGEX-Lys012WT	1-495	Lys012-1Fw	Lys012-495Rv	pGEX-6P-2
pGEX-Lys012Δmt1	161-495	Lys012-161Fw	Lys012-495Rv	pGEX-6P-2
pGEX-Lys012Δmt2	1-221, 390-495	1-221; Lys012-1Fw	1-221; Lys012-Δmt2Rv	pGEX-6P-2
		390-495; Lys012-Δmt2Fw	390-495; Lys012-495Rv	
		Overwrap; Lys012-1Fw	Overwrap; Lys012-495Rv	
pGEX-Lys012Δmt2′	1-221	Lys012-1Fw	Lys012-221Rv	pGEX-6P-2
pGEX-Lys012Δmt3	1-408	Lys012-1Fw	Lys012-408Rv	pGEX-6P-2
pGEX-Lys012Δmt4	386-495	Lys012-386Fw	Lys012-495Rv	pGEX-6P-2
pGEX-Lys012Δmt5	161-408	Lys012-161Fw	Lys012-408Rv	pGEX-6P-2
pGEX-Lys012Δmt6	1-187	Lys012-1Fw	Lys012-187Rv	pGEX-6P-2
**Primers**	**Sequences**			
Lys012-1Fw	5′-TCCCCAGGAATTCCCATGGCTAAGACTCAAGCAGA-3′			
Lys012-161Fw	5′-TCCCCAGGAATTCCCATGATACCTGTAAAAGCAGGAA-3′			
Lys012-386Fw	5′-TCCCCAGGAATTCCCATGACAAGTAGCGCA-3′			
Lys012-187Rv	5′-CGCTCCAGTCGACCCCTATTTCTTTTTAGGTGCAG-3′			
Lys012-221Rv	5′-CGCTCGAGTCGACCCCTATGAAGAACGACCTGC-3′			
Lys012-408Rv	5′-CGCTAGTCGACCCCTAAGTTCCGTACTGGTTC-3′			
Lys012-495Rv	5′-CGCTCGAGTCGACCCCTACTTGAATACTCCCCAGG-3′			
Lys012-Δmt2Fw	5′-CACAACGATGCAGGTCGTTCTTCAAGTACACCGGCAACTAGACCAGTTAC-3′			
Lys012-Δmt2Rv	5′-GTAACTGGTCTAGTTGCCGGTGTACTTGAAGAACGACCTGCATCGTTGTG-3′			

Sequences complementary to linearized pGEX-6P-2 for In-fusion cloning are underlined.

**Table 3:** Protein structure modeling information.

	Homologous Protein	Source Organism	Residues	Protein ID in PDBe	Seq. Identity	Ligands
Lys-phiSA012 CHAP	ORF30/31	*Staphylococcus* virus K	1–165	4ct3.1	99.39%	Ca^2+^
	CHAP domain	(Gene name: PhageK_071)				Cl^−^
						Hg^2+^
Lys-phiSA012 AMID	Endolysin	*Staphylococcus* phage GH15	165–403	4ols	100%	Zn^2+^
	*N*-acetylmuramoyl-l-alanine amidase	(Gene name: GH15_071)				Mg^2+^
						Fe^3+^

**Table 4:** LysK-like endolysins.

Source of Endolysin	Query Cover (Nucleotide)	Identity (Nucleotide)	Accession	Resideu at Position (Amino Asid)																							Identity (Amino Acid)
				26	48	83	107	109	113	165	231	300	372	380	383	406	414	425	437	452	453	470	484	485	486	493	
Bacteriophage K	100%	100%	AY176327.1	V	G	S	F	S	E	A	N	A	D	V	D	Y	N	V	V	V	C	N	N	Q	I	V	100.00%
Bacteriophage JD007	100%	99%	JX878671.1	V	G	S	F	S	E	A	N	A	D	V	D	Y	N	V	V	V	C	N	N	Q	I	V	100.00%
Bacteriophage SA012	100%	99%	AB903967.1	V	G	N	F	S	E	A	N	A	D	V	D	Y	N	V	V	V	C	N	N	Q	I	V	99.80%
Bacteriophage vB_Sau_CG	100%	99%	KY794641.1	V	G	N	F	S	Q	A	T	A	D	V	D	Y	N	V	V	V	C	N	N	Q	I	V	99.39%
Bacteriophage S25-3	100%	99%	AB853330.1	V	G	N	F	S	E	E	N	T	D	V	D	Y	N	V	V	V	C	N	N	Q	I	V	99.39%
Bacteriophage S25-4	100%	99%	AB853331.1	V	G	N	F	S	E	E	N	T	D	V	D	Y	N	V	V	V	C	N	N	Q	I	V	99.39%
Bacteriophage SA3	100%	96%	MF001365.1	I	G	S	F	S	Q	A	N	A	D	V	D	Y	N	V	V	V	C	N	N	H	I	V	99.39%
Bacteriophage qdsa002	100%	96%	KY779849.1	I	G	S	F	S	Q	A	N	A	D	V	D	Y	N	V	V	V	C	N	N	H	I	V	99.39%
Bacteriophage GH15	100%	96%	JQ686190.1	I	G	S	F	S	Q	A	N	A	D	V	D	Y	N	V	V	V	C	D	N	H	I	V	99.19%
Bacteriophage vB_Sau_Clo6	100%	94%	KY794642.1	V	K	N	Y	E	E	A	K	A	N	V	K	Y	N	V	V	I	C	D	S	Y	T	I	97.98%
Bacteriophage vB_Sau_S24	100%	94%	KY794643.1	V	K	N	Y	E	E	A	K	A	N	V	K	Y	N	V	V	V	A	N	N	Q	V	T	97.37%
Bacteriophage MCE-2014	99%	99%	KJ888149.1	V	G	S	F	S	E	A	N	A	D	V	D	Y	N	V	V	V	C	N	S	H	V	V	99.39%
Bacteriophage IPLA-RODI	99%	95%	KP027446.1	I	G	S	F	S	Q	A	N	A	D	I	D	F	S	I	I	V	C	N	N	H	I	V	98.38%
					**CHAP**						**Amidase**						**SH3b**										

Amino acid changes compared to Lys-phiK are indicated in blue (Lys-phiSA012) or yellow (other).

## Supplementary Materials

The following are available online at http://www.mdpi.com/1424-8247/11/1/25/s1.
