# Supplementary figures and images for: Characterization of the Lytic Capability of a LysK-Like Endolysin, Lys-phiSA012, Derived from a Polyvalent Staphylococcus aureus Bacteriophage
Source: Pharmaceuticals (Basel). 2018 Feb 24;11(1):25. doi: 10.3390/ph11010025 (PMC5874721; doi:10.3390/ph11010025)

# Supplementary information

## Fig. S1

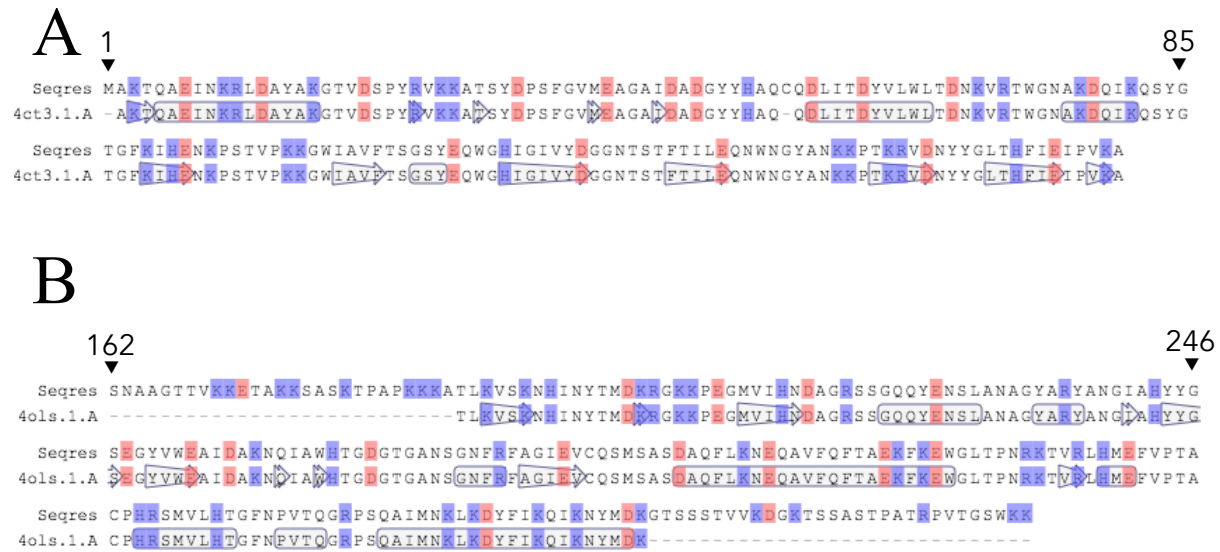

# Supplementary information

## Fig. S2

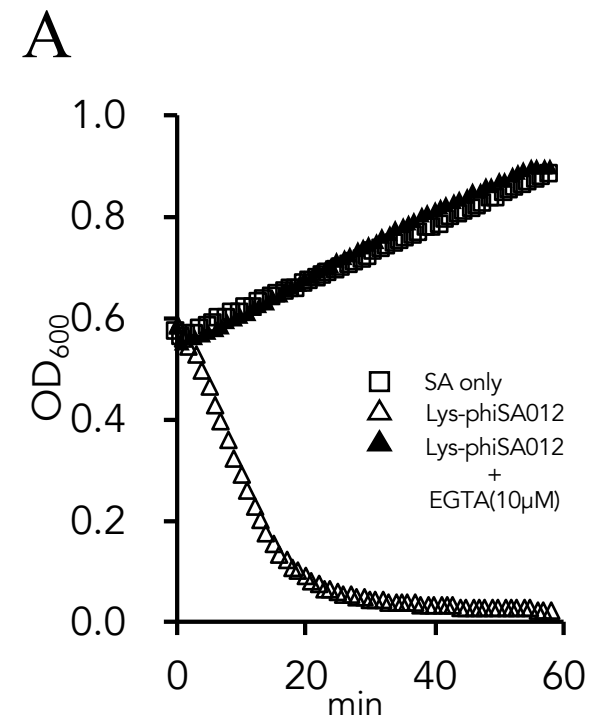

Supplement: Supplementary file 1 [file pharmaceuticals-11-00025-s001.pdf]
